# Supplementary material for: Isolation and Characterization of Novel Bacteriophages to Target Carbapenem-Resistant Acinetobacter baumannii
Source: Antibiotics (Basel). 2024 Jun 29;13(7):610. doi: 10.3390/antibiotics13070610 (PMC11273472; doi:10.3390/antibiotics13070610)
Supplement: Supplementary file 1 [file antibiotics-13-00610-s001.zip › antibiotics-3053644-supplementary.pdf]

antibiotics

MDPI

Supplementary Materials

Isolation and Characterization of Novel Bacteriophages to Target Carbapenem-Resistant *Acinetobacter baumannii*

Yoon-Jung Choi †, Shukho Kim †, Minsang Shin and Jungmin Kim *

Department of Microbiology, School of Medicine, Kyungpook National University, 41944 Daegu, Republic of Korea; yjchoi8727@knu.ac.kr (Y.-J.C.); shukhokim@knu.ac.kr (S.K.); shinms@knu.ac.kr (M.S.)
* Correspondence: minkim@knu.ac.kr; Tel.: +82-53-420-4845
† These authors contributed equally to this work.

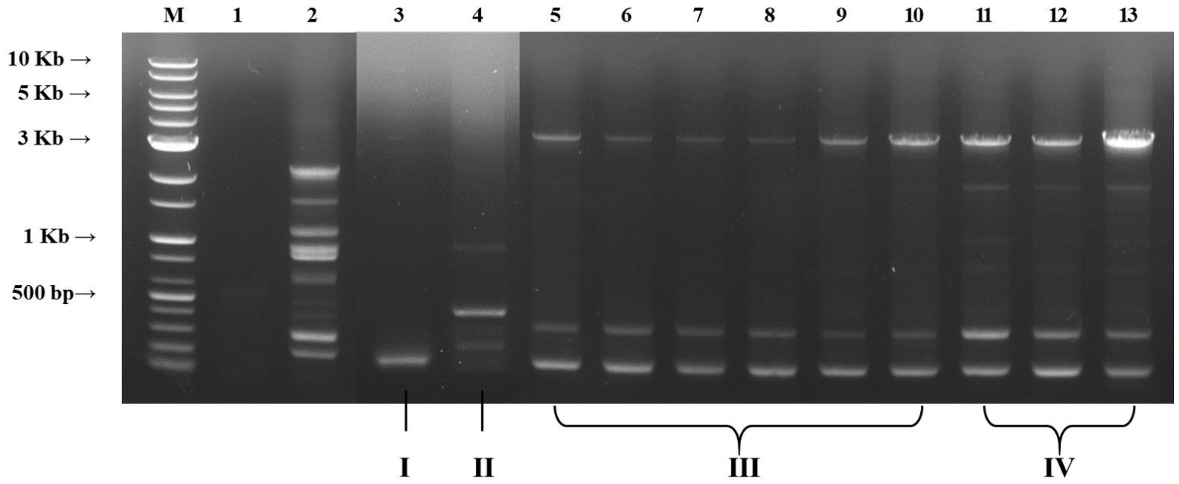

Figure S1. Agarose gel electrophoresis showing RAPD–PCR DNA amplification from isolated carbapenem-resistant *Acinetobacter baumannii* specific phages. The primers employed in this study included OPL5 (5′-ACGCAGGCAC-3′), P1 (5′-CCGCAGCCAA-3′), and P2 (5′-AACGGGCAGA-3′); each was tested at a concentration of 10 pM. The homology of the PCR products was validated using 1% agarose gel electrophoresis. M: Kb ladder, lane 1: sterilized water used as the negative control, lane 2: ϕ1656-2 used as the positive control, lane 3: AW8, lane 4: AW9, lanes 5–8: W15-1–AW15-4, lanes 10–13: AW16-1–AW16-5 with three primer mixes (P1, P2, and OPL5).; Table S1: Bacteriophage efficiency of plating (EOP).

Table S1. Bacteriophages efficiency of plating (EOP).

CRAB	ST	Acinetobacter phage vB_AbaP_W8	Acinetobacter phage vB_AbaSi_W9	Acinetobacter phage vB_AbaSt_W16
KBN10P02782	552	-	-	1.00
LIS20145805	229	0.84 ± 0.125	-	0.97 ± 0.167
LIS20145719		0.85 ± 0.154	-	0.84 ± 0.188
LIS20144539		0.88 ± 0.175	-	0.94 ± 0.095
KBN10P04598		0.65 ± 0.157	-	0.91 ± 0.104
LIS20130976	357	-	0.084 ± 0.013	0.54 ± 0.124
LIS20130721		-	0.064 ± 0.009	0.64 ± 0.154
LIS20130567		-	0.075 ± 0.011	0.71± 0.108
KBN10P04322		-	0.034 ± 0.005	0.09 ± 0.017
KBN10P05102	784	0.94 ± 0.108	0.88 ± 0.171	0.75 ± 0.184
KBN10P04703		0.84 ± 0.154	0.67 ± 0.161	0.84 ± 0.111

KBN10P04697		0.74 ± 0.110	0.71 ± 0.094	0.88 ± 0.097
KBN10P02972	191	-	0.024 ± 0.004	-
KBN10P02901		-	0.011 ± 0.008	-
KBN10P02755		-	-	-
KBN10P04594		-	-	-
KBN10P04627		-	0.021 ± 0.009	-
KBN10P04948		-	0.011 ± 0.008	-
KBN10P02768	208	-	-	-
LIS20132370		-	-	-
KBN10P04322		-	0.002 ± 0.001	-
LIS20140444	369	-	0.034 ± 0.011	-
LIS20138989		-	0.084 ± 0.002	-
LIS20137924		-	0.061 ± 0.017	-
KBN10P04633		-	0.052 ± 0.008	-
KBN10P05663		-	0.38 ± 0.011	-
KBN10P05982		-	0.055 ± 0.015	-
KBN10P04600	451	-	0.088 ± 0.022	-
KBN10P05231		-	0.074 ± 0.027	-
A. baumannii ATCC17978		1.00	1.00	0.30 ± 0.054
A. baumannii ATCC19606		-	0.001 ± 0.001	-
